# 371. Clinical Characteristics and Prognoses for Carbapenem-Resistant Bacterial Infections in Patients with Cancer in Japan: A Multicenter Study from the Multi-Drug Resistant Organisms Clinical Research Network (MDRnet)

**DOI:** 10.1093/ofid/ofad500.441

**Published:** 2023-11-27

**Authors:** Naoya Itoh, Takanori Kawabata, Nana Akazawa, Kayoko Hayakawa, Masahiro Suzuki, Aki Sakurai, Kohei Uemura, Yasufumi Matsumara, Ryota Hase, Hideaki Kato, Takehiro Hashimoto, Takashi Matono, David van Duin, Norio Ohmagari, Yohei Doi, Sho Saito

**Affiliations:** Aichi Cancer Center, Nagoya, Aichi, Japan; National Cerebral and Cardiovascular Center, Suita, Osaka, Japan; Aichi Cancer Center, Nagoya, Aichi, Japan; National Center for Global Health and Medicine, Shinjuku-ku, Tokyo, Japan; Fujita Health University School of Medicine, Toyoake, Aichi, Japan; University of Texas Health Science Center, McGovern Medical School, Houston, TX; The University of Tokyo, Bunkyo-ku, Tokyo, Japan; Kyoto University Graduate School of Medicine, Kyoto, Kyoto, Japan; Japanese Red Cross Narita Hospital, Narita-shi, Chiba, Japan; Yokohama City University Hospital, yokohama-shi, Kanagawa, Japan; Oita University Hospital, Yufu-shi, Oita, Japan; Aso Iizuka Hospital, Iizuka, Fukuoka, Japan; University of North Carolina at Chapel Hill, Chapel Hill, NC; National Centre for Global Health and Medicine, Shinjuku, Tokyo, Japan; Fujita Health University School of Medicine, Toyoake, Aichi, Japan; National Center for Global Health and Medicine, Shinjuku-ku, Tokyo, Japan

## Abstract

**Background:**

Carbapenem-resistant bacteria in patients with cancer are concerning due to the high risk of infection and mortality rates; however, the characteristics and prognoses of carbapenem-resistant infections in this population are currently unknown in Japan. We hence investigated the features and outcomes of carbapenem-resistant bacterial infections (CRBI) in patients with cancer in Japan.

**Methods:**

From April 1, 2019, to March 31, 2022, patients with CRBI who either had cancer or no cancer were prospectively enrolled at eight centers as part of the Multi-Drug Resistant Organisms Clinical Research Network (MDRnet). The primary outcome was the 30-day all-cause mortality rates in patients with and without cancer. Two secondary outcomes were evaluated: 1) composite outcomes including mortality, worsening of clinical course after culture collection, intensive care unit stay, intubation, new dialysis from the date of culture collection to the end of antimicrobial therapy, and readmission within 90 days after discharge; and 2) the length of hospital stay after CRBI excluding death.

**Results:**

We included a total of 167 patients, with 66 (39.5%) in the cancer group and 101 (60.5%) in the non-cancer group. The 30-day mortality rates in the cancer and non-cancer groups were 18.2% (12/66) and 14.0% (14/101), respectively (p = 0.45), while the composite outcomes in the cancer and non-cancer groups were 56.1% (37/66) and 43.6% (44/101), respectively (p = 0.12). Average duration of hospitalization was not significantly different between the two groups (cancer group, 44.6 days; non-cancer group, 51.0 days; p = 0.55). Propensity score analysis using inverse probability weighting also showed no significant difference in 30-day mortality and average duration of hospitalization (p = 0.22 and 0.98, respectively); however, the composite outcome was significantly higher in the cancer group than in non-cancer controls (odds ratio, 2.41; 95% confidence interval, 1.11–5.21; p = 0.03).

**Figure:**
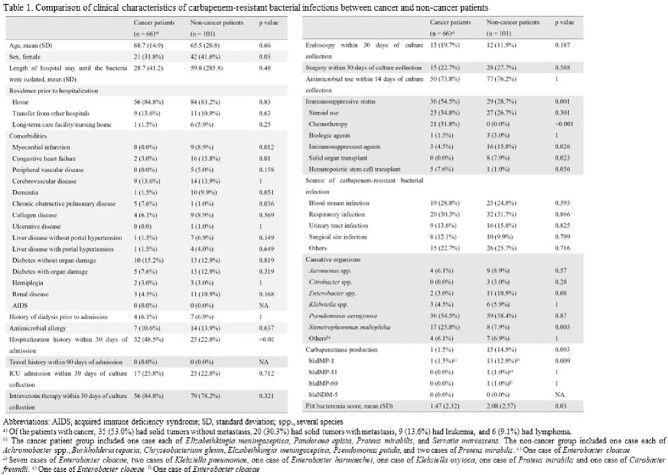

**Figure:**
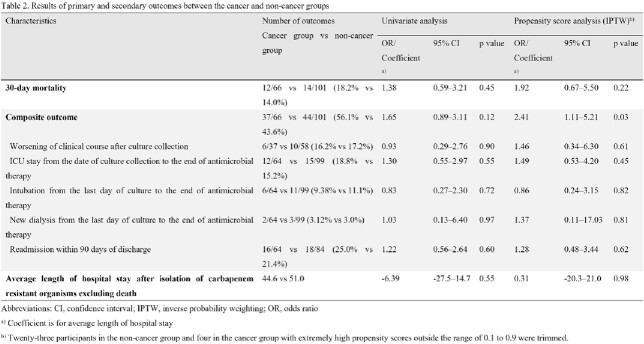

**Conclusion:**

There was no difference in 30-day mortality rates between the cancer and non-cancer patient groups; however, we found a significant difference in the composite outcome. Patients with cancer who had CRBI experienced a worse clinical course that non-cancer patients.

**Disclosures:**

**Masahiro Suzuki, PhD**, KANTO Chemical co., inc.: Grant/Research Support **Yasufumi Matsumara, MD, PhD**, Beckman Coulter: Grant/Research Support|Presicion System Science: Grant/Research Support|Toyobo: Grant/Research Support **David van Duin, MD, PhD**, Entasis: Advisor/Consultant|Merck: Advisor/Consultant|Merck: Grant/Research Support|Pfizer: Advisor/Consultant|Pfizer: Honoraria|Qpex: Advisor/Consultant|Roche: Advisor/Consultant|Shionogi: Advisor/Consultant|Shionogi: Grant/Research Support|Union: Advisor/Consultant|Utility: Advisor/Consultant **Yohei Doi, MD, PhD**, bioMerieux: Advisor/Consultant|FujiFilm: Advisor/Consultant|Gilead: Advisor/Consultant|Gilead: Honoraria|GSK: Advisor/Consultant|Meiji Seika Pharma: Advisor/Consultant|Moderna: Advisor/Consultant|Moderna: Honoraria|MSD: Advisor/Consultant|MSD: Honoraria|Shionogi: Advisor/Consultant|Shionogi: Grant/Research Support|Shionogi: Honoraria **Sho Saito, MD, PhD**, Shionogi & Company, Limited: Grant/Research Support

